# Discovery of imatinib-responsive *FIP1L1-PDGFRA* mutation during refractory acute myeloid leukemia transformation of chronic myelomonocytic leukemia

**DOI:** 10.1186/1756-8722-7-26

**Published:** 2014-03-27

**Authors:** Shilpan Shah, Sanam Loghavi, Guillermo Garcia-Manero, Joseph D Khoury

**Affiliations:** 1 Department of Leukemia, The University of Texas MD Anderson Cancer Center, 1515 Holcombe Boulevard, MS-72, 77030 Houston, TX, USA; 2 Department of Hematopathology, The University of Texas MD Anderson Cancer Center, 1515 Holcombe Boulevard, MS-72, 77030 Houston, TX, USA

**Keywords:** *FIP1L1-PDGRFA*, Chronic myelomonocytic leukemia, Imatinib mesylate

## Abstract

The *FIP1L1-PDGFRA* rearrangement results in constitutive activation of the tyrosine kinase PDGFRA. Neoplasms harboring this rearrangement are responsive to imatinib mesylate at doses much lower than those recommended for the treatment of chronic myelogenous leukemia. Only a single report has described the identification of *FIP1L1-PDGFRA* in chronic myelomonocytic leukemia (CMML). Herein, we present a case report of a patient in whom the *FIP1L1-PDGFRA* was discovered as he evolved from CMML to acute myeloid leukemia (AML). The presence of a dominant neoplastic clone with *FIP1L1-PDGFRA* rearrangement was suspected on the basis of sudden onset of peripheral and bone marrow eosinophilia and confirmed by fluorescence in situ hybridization and molecular diagnostic tests. Whereas the patient was initially refractory to chemotherapy before the rearrangement was detected, subsequent therapy with imatinib led to complete remission.

## Background

Hypereosinophilia is a feature of a variety of uncommon hematologic disorders like hyperseosinophilic syndrome (HES), systemic mastocytosis (SM) and chronic eosinophilic leukemia (CEL). Approximately 4% of patients with HES or SM have interstitial deletion of chromosome 4q12 leading to juxtaposition of *FIP1L1* and *PDGFRA*[1]. The fusion product is exquisitely sensitive to therapy with imatinib mesylate, and hence its identification has important therapeutic ramifications particularly in hematologic disorders presenting with hypereosinophilia [2-5]. We herein present the case of a patient in whom *FIP1L1-PDGFRA* was discovered at the time of evolution from chronic myelomonocytic leukemia (CMML) to refractory acute myeloid leukemia and how therapy with imatinib resulted in durable complete remission.

## Case presentation

A 64-year-old Caucasian man presented to our institution with a 6-month history of progressive leukocytosis. Per the patient’s outside medical records, a bone marrow biopsy at initial presentation had shown a 100% cellular marrow with marked myeloid hyperplasia. Conventional cytogenetics demonstrated a diploid male karyotype. Fluorescence in situ hybridization studies (FISH) were negative for myelodysplasia-associated abnormalities. Molecular studies were negative for *BCR-ABL* rearrangement, and *JAK2*^*V617F*^ and *MPL*^*W515L*^ mutations. Based on these features, he was diagnosed with myelodysplastic/myeloproliferative neoplasm, unclassifiable, and was started on hydroxyurea, 1.5 grams daily. At presentation to our institution, his white blood cell (WBC) count was 44.6 ×10^9^/L with 76% neutrophils, 6% metamyelocytes, 10% monocytes, 6% lymphocytes, 1% eosinophils and 1% blasts, with absolute monocytosis (4.46 × 10^9^/L) and eosinophilia (0.45 × 10^9^/L). He was anemic (hemoglobin 9.0 g/dL) and mildly thrombocytopenic (platelet count 119 × 10^9^/L). He did not have splenomegaly on physical examination. Bone marrow evaluation performed at presentation to our institution revealed a 100% cellular bone marrow with myeloid hyperplasia. Megakaryocytes were decreased in number and included rare dysplastic forms. Wright-Giemsa stained smears prepared from the bone marrow aspirate were remarkable for increased myeloid cells and trilineage dysplasia; a 500-cell differential count showed mildly increased monocytes (6%) and myeloid blasts (7%). The constellation of findings was diagnostic of chronic myelomonocytic leukemia (CMML-1). Conventional cytogenetics showed trisomy 8 in two of twenty analyzed metaphases; this was confirmed by fluorescence in situ hybridization (FISH) using an alpha-satellite (D8Z2) CEP8 probe (positive in 4% of the cells studied). Reverse transcriptase polymerase chain reaction (RT-PCR) performed on the bone marrow aspirate was negative for *BCR-ABL* fusion. Targeted next-generation sequencing mutation analysis was negative for 53 “hotspot” mutations analyzed as described previously [6].

**Figure 1:**
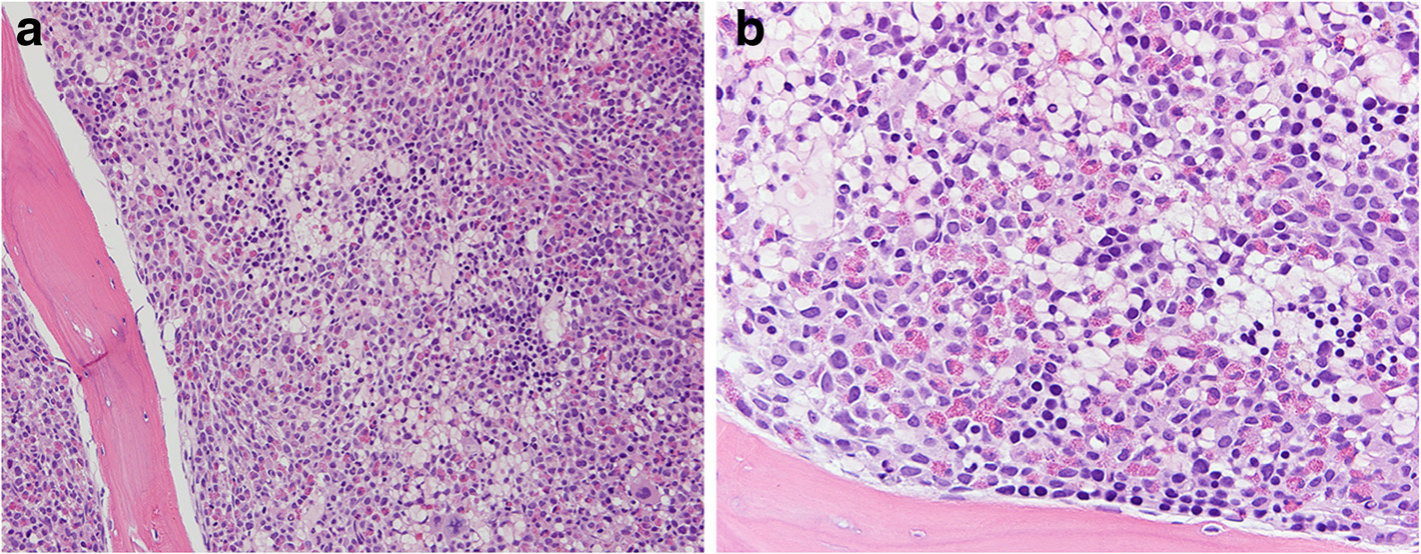
**Bone marrow core biopsy at the time of initial *FIP1L1- PDGFRA *rearrangement discovery.** The bone marrow is hypercellular (100%), with prominent eosinophilia and myeloid hyperplasia, mild increase in immature cells, and features of myelofibrosis manifesting primarily as cellular streaming. (**a**: 10× objective; **b**: 20× objective; hematoxylin and eosin stain).

The patient was enrolled on the SGI-110 clinical trial, a phase 1–2 dose escalation, multicenter study of SGI-110, a DNA hypomethylating agent, in subjects with intermediate or high-risk myelodysplastic syndromes (MDS) or AML. He received two courses of SGI-110, after which he experienced rapidly progressive leukocytosis with a peak WBC count of 126 × 10^9^/L and new onset of peripheral eosinophilia (11%). Bone marrow evaluation performed at this time demonstrated nearly 100% cellularity with myeloid hyperplasia; in contrast to the previous biopsy, prominent eosinophilia and moderate myelofibrosis were noted in this sample (Figure 1a-b). Wright-Giemsa stained smears showed increased granulocytes with left-shifted maturation and prominent eosinophilia (16%) in a background of trilineage dysplasia. There was no significant increase in bone marrow monocytes (5%). Myeloid blasts comprised 12% of total nucleated cells. The unexpected and abrupt presence of prominent eosinophilia in the peripheral blood and bone marrow at this point in time prompted us to evaluate for *PDGFRA* rearrangement. FISH analysis performed using a LSI-4q12 tricolor rearrangement probe that hybridizes to the chromosome 4q12 region containing the *FIP1L1*, *CHIC2* and *PDGFRA* genes revealed deletion of the *CHIC2* gene in 86.5% of the cells analyzed indicating the presence of the *FIP1L1-PDGFRA* rearrangement. The *FIP1L1-PDGFRA* fusion transcript was further confirmed by RT-PCR. Low-level trisomy 8 was also detected by FISH in this sample. Based on these findings a diagnosis of myelodysplastic/myeloproliferative neoplasm with eosinophilia and *PDGFRA* rearrangement was rendered. A follow-up bone marrow biopsy after one month showed acute myeloid leukemia with 26% blasts. In addition to persistent low-level trisomy 8, conventional cytogenetics and FISH demonstrated a new clone with *TP53* gene deletion. FISH was positive for deletion of the *CHIC2* gene, *TP53* deletion and trisomy 8 in 90%, 10% and 9% of the analyzed cells, respectively.

The patient was then started on imatinib mesylate 400 mg daily along with a short course of idarubucin and subcutaneous cytarabine for cytoreduction. He achieved complete hematologic and morphologic remission and went on to receive a matched unrelated allogeneic stem cell transplant (SCT). To date, the patient remains in complete hematologic, morphologic and molecular remission with successful engraftment as demonstrated by chimerism studies. The sequence of events is provided in Table 1.

**Table 1:** Sequence of clinical events

**Event sequence**	**WBC (×10**^**9**^**)**	**Eosinophils in PB (%, AEC)**	**Eosinophils in BM (%)**	**Blasts in BM (%)**	**Diagnosis**	**Intervention**	**Disease status**
Initial presentation	44.6	1, 0.45	1	7	CMML-1	SGI-110	Refractory
After 2 months	65.1	9, 5.8	5	4	CMML-1	SGI-110	Progression
After 1 month	126	11, 13.8	16	12	Myelodysplastic/myeloproliferative neoplasm with eosinophilia, and *PDGFRA* rearrangement	Fludarabine + Cytarabine	Progression
After 1 month	6.4	1, 0.06	1	26	AML with *PDGFRA* rearrangement	Imatinib (with Idarubicin + Cutarabine)	Remission

WBC: white blood cells, PB: peripheral blood, AEC: absolute eosinophil count, BM: bone marrow, CMML: chronic myelomonocytic leukemia, AML: acute myeloid leukemia.

## Discussion

The PDGFRA and PDGFRB proteins are members of the class III receptor kinase family that also includes c-KIT, and FLT3 [7]. *PDGFRA* is located on chromosome 4q12 [8]. A small interstitial deletion of 4q12 leads to juxtaposition of *FIP1L1* and *PDGFRA* resulting in a gain of function fusion protein with signal independent kinase activity and therefore increased cell proliferation and survival [9]. This interstitial deletion is generally cryptic and not detectable using standard cytogenetic banding techniques.

*FIP1L1- PDGFRA* rearrangements are often associated with chronic eosinophilic leukemia and hypereosinophilic syndromes [9,10] as well as systemic mastocytosis [11]. Pardanani *et al.* reported a prevalence of approximately 4% for *FIP1L1- PDGFRA* fusion gene in a large series of patients with suspected or established HES or systemic mastocysosis [1]. We recently described a case of chronic neutrophilic leukemia associated with *FIP1L1- PDGFRA* rearrangement [4]. The basis for the apparent lineage predilection of *FIP1L1- PDGFRA* for eosinophils is not well understood. The hypothesis is that it is present in all myeloid lineages, but that eosinophils are particularly sensitive to the *FIP1L1- PDGFRA* proliferative signal [12]. In contrast to rearrangements involving *PDGFRB,* the *FIP1L1- PDGFRA* rearrangement is exceedingly rare in the setting of chronic myelomonocytic leukemia [13-16]. To our knowledge, only one other case has been reported in the literature by Zota *et al*. [17] In contrast to our patient, the patient reported by Zota *et al.* did not evolve in to acute myeloid leukemia, albeit the acquisition of *FIP1L1- PDGFRA* fusion was considered a feature of disease evolution. Initiation of imatinib resolved eosinophilia but did not effectively improve other counts, and the patient subsequently progressed to CMML-2 and developed extramedullary disease in abdominal lymph nodes; she succumbed in 10 months. A case series from Germany described five patients with *FIP1L1- PDGFRA* who presented with AML and eosinophilia, but no history of antecedent myeloid malignancy was reported for any of the patients [18]. A summary of case reports describing *PDGFRA* rearrangements arising in patients with myeloid neoplasms commonly not associated with such rearrangements is provided in Table 2. All patients received imatinib therapy and achieved at least a hematologic response. One patient got sorafenib and another got dasatinib after acquiring resistance to imatinib. Two of eight patients maintained molecular response, while two maintained hematologic response at last reported follow up. Notably, clonal acquisition of *FIP1L1-PDGFRA* has not been reported in the setting of acute myeloid leukemia evolving from CMML.

**Table 2:** ***PDGFRA *** **rearrangement in unusual adult myeloid neoplasms**

**Author/journal**	**Disease**	**Treatment**	**Outcome**
Tang et al., Acta Haematol 2012;128:83–87	Myeloproliferative neoplasm with eosinophilia	Imatinib – started at 400 mg daily and maintained at 100 mg daily	Complete hematologic and molecular remission at 12 months
Papanikolaou et al., Ann Hematol. 2012 May;91(5):785-7	Chronic eosinophilic leukemia with lytic bone lesions	Imatinib 200 mg daily; on progression, nilotinib 400 mg BID	Progressed to erythroblastoid blast crisis after 2 years on TKI
Sorour et al., Br J Haematol. 2009 Jul;146(2):225-7	Acute myeloid leukemia with eosinophilia	Imatinib with FLAG-Ida followed by matched unrelated allograft	Relapsed with Imatinib resistance; started on Dasatinib but died 15 months later
Lierman et al., Leukemia. 2009 May;23(5):845-51	Chronic eosinophilic leukemia blast crisis with Imatinib resistance	Sorafenib 400 mg BID	Hematologic response without molecular response for 3 months
Zota et al., J Clin Oncol. 2008 Apr 20;26(12):2040-1	Chronic myelomonocytic leukemia	Imatinib 400 mg BID	Resolution of eosinophilia without other hematologic response; progressed to extramedullary disease
Florian et al., Leuk Res. 2006 Sep;30(9):1201-5	Systemic mastocytosis with chronic eosinophilic leukemia	Incomplete response to hydroxyurea, corticosteroids and interferon-alpha; started on imatinib 100 mg in 2002	Long-term response to low-dose Imatinib (50-100 mg) after inadequate responses to previous therapies
Von Bubnoff et al., Leukemia. 2005 Feb;19(2):286-7	Chronic myeloproliferative disorder with eosinophilia	Imatinib 100-400 mg daily	Hematologic and symptomatic response for 6 months; progression to myeloid blasts crisis and malignant pleural effusion
Saflet et al., Genes Chromosomes Cancer 2004 May;40(1):44-50	Atypical chronic myeloid leukemia with t(4;22) leading to formation of BCR-PDGFRA fusion gene	Imatinib 100 mg daily	Complete hematologic response at 7-month follow up

Our case highlights the importance of assessing for *PDGFRA* rearrangement in myeloid neoplasms with *de novo* or subsequently acquired eosinophilia. The identification of the *FIP1L1- PDGFRA* fusion gene is significant since imatinib has excellent efficacy at low doses (100-400 mg daily) in *FIP1L1-PDGFRA*-positive neoplasms [2,5]. Of note, due to the 250-fold lower IC_50_ as compared to *BCR-ABL*, reports suggest that even once weekly doses of imatinib are adequate in the setting of *FIP1L1-PDGFRA*[3]. However, these responses are eventually lost due to emergence of an imatinib-resistant T614I mutation in the ATP-binding site of *PDGFRA*[9]. In our patient, the ability to induce a complete remission using imatinib at a time when the patient was unresponsive to chemotherapy induction permitted subsequent allogeneic SCT and an ensuing durable remission as of last follow up.

## Conclusion

We describe a case report of a patient who transformed from CMML to AML which was refractory to standard chemotherapy. Emergence of peripheral and bone marrow hypereosinophilia during this transformation led to suspicion of presence of *FIP1L1-PDGFRA* rearrangement, which was confirmed by FISH and RT-PCR. Treatment with imatinib led to a complete remission and permitted allogeneic SCT therapy.

## Endnote

The identification of new onset of eosinophilia in acute myeloid leukemia arising in a patient with chronic myelomonocytic leukemia might indicate acquisition of imatinib-responsive *FIP1L1-PDGFRA* rearrangement.

## Consent

Granted under protocol approved by the Institutional Review Board of The University of Texas M.D. Anderson Cancer Center.

## Abbreviations

CMML: Chronic myelomonocytic leukemia; AML: Acute myeloid leukemia; HES: Hyperseosinophilic syndrome; SM: Systemic mastocytosis; CEL: Chronic eosinophilic leukemia; FISH: Fluorescence in situ hybridization studies; PCR: Polymerase chain reaction; MDS: Myelodysplastic syndromes.

## Competing interests

The authors declare no competing interest pertaining related to this study.

## Authors’ contributions

SS, GGM and JDK: conception of manuscript, chart review, and manuscript preparation; SL: chart review and manuscript preparation. All authors have read and approved the final manuscript.

## References

[B1] Pardanani A Ketterling RP Li CY Patnaik MM Wolanskyj AP Elliott MA Camoriano JK Butterfield JH Dewald GW Tefferi A FIP1L1-PDGFRA in eosinophilic disorders: prevalence in routine clinical practice, long-term experience with imatinib therapy, and a critical review of the literature Leuk Res 2006 30 8 965 70 10.1016/j.leukres.2005.11.011 16406016

[B2] Helbig G Stella-Holowiecka B Grosicki S Bober G Krawczyk M Wojnar J Reiter A Hochhaus A Holowiecki J The results of imatinib therapy for patients with primary eosinophilic disorders Eur J Haematol 2006 76 6 535 6 10.1111/j.1600-0609.2006.00652.x 16608506

[B3] Helbig G Stella-Hołowiecka B Majewski M Całbecka M Gajkowska J Klimkiewicz R Moskwa A Grzegorczyk J Lewandowska M Hołowiecki J A single weekly dose of imatinib is sufficient to induce and maintain remission of chronic eosinophilic leukaemia in FIP1L1-PDGFRA-expressing patients Br J Haematol 2008 141 2 200 4 10.1111/j.1365-2141.2008.07033.x 18307562

[B4] Jain N Khoury JD Pemmaraju N Kollipara P Kantarjian H Verstovsek S Imatinib therapy in a patient with suspected chronic neutrophilic leukemia and FIP1L1-PDGFRA rearrangement Blood 2013 122 19 3387 8 10.1182/blood-2013-07-516500 24203930

[B5] Metzgeroth G Walz C Erben P Popp H Schmitt-Graeff A Haferlach C Fabarius A Schnittger S Grimwade D Cross NC Hehlmann R Hochhaus A Reiter A Safety and efficacy of imatinib in chronic eosinophilic leukaemia and hypereosinophilic syndrome: a phase-II study Br J Haematol 2008 143 5 707 15 10.1111/j.1365-2141.2008.07294.x 18950453

[B6] Alayed K Patel KP Konoplev S Singh RR Routbort MJ Reddy N Pemmaraju N Zhang L Shaikh AA Aladily TN Jain N Luthra R Medeiros LJ Khoury JD TET2 mutations, myelodysplastic features, and a distinct immunoprofile characterize blastic plasmacytoid dendritic cell neoplasm in the bone marrow Am J Hematol 2013 88 12 1055 61 10.1002/ajh.23567 23940084

[B7] Reilly JT Class III receptor tyrosine kinases: role in leukaemogenesis Br J Haematol 2002 116 4 744 57 10.1046/j.0007-1048.2001.03294.x 11886377

[B8] Gronwald RG Adler DA Kelly JD Disteche CM Bowen-Pope DF The human PDGF receptor alpha-subunit gene maps to chromosome 4 in close proximity to c-kit Hum Genet 1990 85 3 383 5 1697560 10.1007/BF00206767

[B9] Cools J DeAngelo DJ Gotlib J Stover EH Legare RD Cortes J Kutok J Clark J Galinsky I Griffin JD Cross NC Tefferi A Malone J Alam R Schrier SL Schmid J Rose M Vandenberghe P Verhoef G Boogaerts M Wlodarska I Kantarjian H Marynen P Coutre SE Stone R Gilliland DG A tyrosine kinase created by fusion of the PDGFRA and FIP1L1 genes as a therapeutic target of imatinib in idiopathic hypereosinophilic syndrome N Engl J Med 2003 348 13 1201 14 10.1056/NEJMoa025217 12660384

[B10] Pardanani A Brockman SR Paternoster SF Flynn HC Ketterling RP Lasho TL Ho CL Li CY Dewald GW Tefferi A FIP1L1-PDGFRA fusion: prevalence and clinicopathologic correlates in 89 consecutive patients with moderate to severe eosinophilia Blood 2004 104 10 3038 45 10.1182/blood-2004-03-0787 15284118

[B11] Pardanani A Pardanani A Ketterling RP Brockman SR Flynn HC Paternoster SF Shearer BM Reeder TL Li CY Cross NC Cools J Gilliland DG Dewald GW Tefferi A CHIC2 deletion, a surrogate for FIP1L1-PDGFRA fusion, occurs in systemic mastocytosis associated with eosinophilia and predicts response to imatinib mesylate therapy Blood 2003 102 9 3093 6 10.1182/blood-2003-05-1627 12842979

[B12] Gotlib J Molecular classification and pathogenesis of eosinophilic disorders: 2005 update Acta Haematol 2005 114 1 7 25 10.1159/000085559 15995322

[B13] Apperley JF Gardembas M Melo JV Russell-Jones R Bain BJ Baxter EJ Chase A Chessells JM Colombat M Dearden CE Dimitrijevic S Mahon FX Marin D Nikolova Z Olavarria E Silberman S Schultheis B Cross NC Goldman JM Response to imatinib mesylate in patients with chronic myeloproliferative diseases with rearrangements of the platelet-derived growth factor receptor beta N Engl J Med 2002 347 7 481 7 10.1056/NEJMoa020150 12181402

[B14] David M Cross NC Burgstaller S Chase A Curtis C Dang R Gardembas M Goldman JM Grand F Hughes G Huguet F Lavender L McArthur GA Mahon FX Massimini G Melo J Rousselot P Russell-Jones RJ Seymour JF Smith G Stark A Waghorn K Nikolova Z Apperley JF Durable responses to imatinib in patients with PDGFRB fusion gene-positive and BCR-ABL-negative chronic myeloproliferative disorders Blood 2007 109 1 61 4 10.1182/blood-2006-05-024828 16960151

[B15] Drechsler M Hildebrandt B Kündgen A Germing U Royer-Pokora B Fusion of H4/D10S170 to PDGFRbeta in a patient with chronic myelomonocytic leukemia and long-term responsiveness to imatinib Ann Hematol 2007 86 5 353 4 10.1007/s00277-006-0247-5 17211520

[B16] La Starza R Rosati R Roti G Gorello P Bardi A Crescenzi B Pierini V Calabrese O Baens M Folens C Cools J Marynen P Martelli MF Mecucci C Cuneo A A new NDE1/PDGFRB fusion transcript underlying chronic myelomonocytic leukaemia in Noonan Syndrome Leukemia 2007 21 4 830 3 17301821 10.1038/sj.leu.2404541

[B17] Zota V Miron PM Woda BA Raza A Wang SA Eosinophilia with FIP1L1-PDGFRA fusion in a patient with chronic myelomonocytic leukemia J Clin Oncol 2008 26 12 2040 1 10.1200/JCO.2007.15.3841 18421057

[B18] Metzgeroth G Walz C Score J Siebert R Schnittger S Haferlach C Popp H Haferlach T Erben P Mix J Müller MC Beneke H Müller L Del Valle F Aulitzky WE Wittkowsky G Schmitz N Schulte C Müller-Hermelink K Hodges E Whittaker SJ Diecker F Döhner H Schuld P Hehlmann R Hochhaus A Cross NC Recurrent finding of the FIP1L1-PDGFRA fusion gene in eosinophilia-associated acute myeloid leukemia and lymphoblastic T-cell lymphoma Leukemia 2007 21 6 1183 8 10.1038/sj.leu.2404662 17377585

